# Sex Differences in β-Adrenergic Responsiveness of Action Potentials and Intracellular Calcium Handling in Isolated Rabbit Hearts

**DOI:** 10.1371/journal.pone.0111411

**Published:** 2014-10-23

**Authors:** Gregory S. Hoeker, Ashleigh R. Hood, Rodolphe P. Katra, Steven Poelzing, Steven M. Pogwizd

**Affiliations:** 1 Departments of Medicine, University of Alabama at Birmingham, Birmingham, Alabama, United States of America; 2 Biomedical Engineering, University of Alabama at Birmingham, Birmingham, Alabama, United States of America; 3 Department of Biomedical Engineering, Virginia Tech Carilion Research Institute, Roanoke, Virginia, United States of America; The Ohio State University, United States of America

## Abstract

Cardioprotection in females, as observed in the setting of heart failure, has been attributed to sex differences in intracellular calcium handling and its modulation by β-adrenergic signaling. However, further studies examining sex differences in β-adrenergic responsiveness have yielded inconsistent results and have mostly been limited to studies of contractility, ion channel function, or calcium handling alone. Given the close interaction of the action potential (AP) and intracellular calcium transient (CaT) through the process of excitation-contraction coupling, the need for studies exploring the relationship between agonist-induced AP and calcium handling changes in female and male hearts is evident. Thus, the aim of this study was to use optical mapping to examine sex differences in ventricular APs and CaTs measured simultaneously from Langendorff-perfused hearts isolated from naïve adult rabbits during β-adrenergic stimulation. The non-selective β-agonist isoproterenol (Iso) decreased AP duration (APD_90_), CaT duration (CaD_80_), and the decay constant of the CaT (τ) in a dose-dependent manner (1–316.2 nM), with a plateau at doses ≥31.6 nM. The Iso-induced changes in APD_90_ and τ (but not CaD_80_) were significantly smaller in female than male hearts. These sex differences were more significant at faster (5.5 Hz) than resting rates (3 Hz). Treatment with Iso led to the development of spontaneous calcium release (SCR) with a dose threshold of 31.6 nM. While SCR occurrence was similar in female (49%) and male (53%) hearts, the associated ectopic beats had a lower frequency of occurrence (16% versus 40%) and higher threshold (100 nM versus 31.6 nM) in female than male hearts (p<0.05). In conclusion, female hearts had a decreased capacity to respond to β-adrenergic stimulation, particularly under conditions of increased demand (i.e. faster pacing rates and “maximal” levels of Iso effects), however this reduced β-adrenergic responsiveness of female hearts was associated with reduced arrhythmic activity.

## Introduction

Stimulation of adrenergic receptors by catecholamines is the predominant regulatory mechanism for cardiovascular performance [1], [2]. Many cardiovascular diseases involve changes in β-adrenergic signaling that often lead to decreased cardiac performance, cardiac arrhythmias, and sudden cardiac death [3], [4]. This has made β-adrenergic receptors (β-ARs), and their signaling cascades, a significant therapeutic target in cardiovascular disease.

Sex differences in β-adrenergic responsiveness have been associated with cardioprotection in females, conferring a survival benefit in female patients with heart failure [5], [6]. Given the association of sympathetic stimulation with arrhythmias and mortality in cardiovascular disease, female cardioprotection and potential sex differences in β-adrenergic responsiveness are of particular interest. Despite this clear need, this important issue has been investigated by a limited number of studies. The results of these studies have been inconsistent and have mostly been limited to studies of contractility, ion channel function, or calcium handling alone. Moreover, there is controversy regarding the existence or nature of sex differences in the effects mediated by β-AR activation [7]–[10]. One factor that could potentially contribute to this lack of agreement is the variability in the dose(s) of β-AR agonists that were used in these studies.

In particular, there is a lack of studies with an integrated assessment of electrical and mechanical cardiac function in the same preparation. Given the complex interaction of transmembrane potential and intracellular calcium handling through the process of excitation-contraction coupling, experiments are needed to explore the relationship between β-AR activation-induced changes in action potentials (APs) and intracellular calcium transients (CaTs). In the present study, we simultaneously optically mapped transmembrane potential (V_m_) and intracellular calcium handling (Ca^2+^) in Langendorff-perfused hearts isolated from naïve female and male adult rabbits to investigate sex differences in ventricular APs and CaTs measured at physiologic temperature and pacing rates, the dose response to the non-selective β-agonist isoproterenol (Iso), and Iso-induced arrhythmias. We hypothesized that ventricular APs and CaTs would show unique profiles of response to β-AR activation, and that the degree of sex differences would vary as a function of the dose of Iso treatment.

## Materials and Methods

### Experimental preparation

All experiments were conducted in compliance with the Guide for the Care and Use of Laboratory Animals published by the U.S. National Institutes of Health (NIH Publication No. 85–23, revised 1996). The protocols were approved by the Institutional Animal Care and Use Committee (IACUC) of the University of Alabama at Birmingham. Studies were conducted in naïve, sexually mature (age 6–9 months, 3.5–4.5 kgs) New Zealand White rabbits of both sexes. See Information S1 for an expanded description of the methods. Hearts were rapidly excised, cannulated, and retrogradely perfused in Langendorff-fashion with a modified Tyrode's solution (in mM: NaCl 128.2, KCl 4.7, NaHCO_3_ 20, NaH_2_PO_4_ 1.19, MgCl_2_ 1.05, glucose 11.1, CaCl_2_ 1.8, albumin 100 mg/L) that was heated and bubbled with 95% O_2_/5% CO_2_ to maintain physiologic temperature (36–37°C) and pH (7.40–7.45). The flow rate was adjusted to maintain a perfusion pressure of 50–70 mmHg.

**Figure 1:**
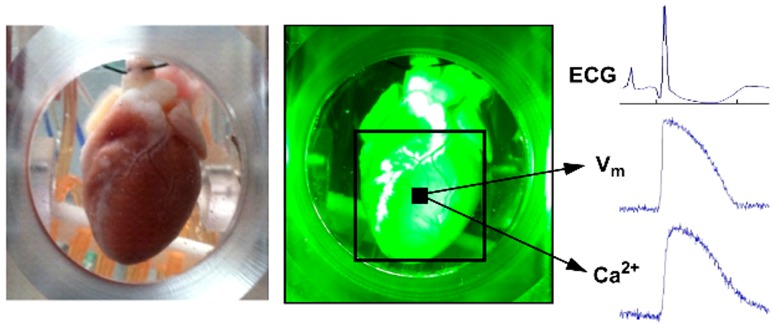
Optical mapping of V_m_ and Ca^2+^ in isolated rabbit hearts. *Left*, Langendorff-perfused isolated rabbit heart mounted in the tissue bath with the anterior surface of the ventricles centered in the viewing window. *Center*, heart illuminated with the high power green (530 nm peak λ) LEDs with the black box representing the field of view. *Right*, representative recordings of the volume-conducted electrocardiogram (ECG), optical AP (V_m_), and CaT (Ca^2+^) measured simultaneously during a single paced beat.

In preliminary studies it was found that treatment with 100 nM Iso dramatically increased the sinus heart rate from approximately 150 beats per minute (2.5 Hz) at baseline to nearly 300 beats per minute (5 Hz), thereby preventing the use of external pacing rates less than 5 Hz (see Information S1 for additional details). To address this issue, complete heart block (CHB) was induced by removing the right atrium (containing the sinus node) and ablating the atrioventricular node using electrocautery [11]. CHB resulted in complete atrio-ventricular dissociation and a junctional rhythm ≤60 beats per min, thereby allowing for testing of a wider range of pacing frequencies and to facilitate detection of ectopic beats during β-AR activation. The heart was then mounted in a custom tissue bath (Figure 1), immersed in the superfusate, and positioned such that the anterior surface of the left ventricle (LV) was visible through a viewing window (soda lime glass). Three evenly spaced electrodes mounted to the walls of the tissue bath were used to record a volume-conducted electrocardiogram (ECG).

The isolated heart was allowed to stabilize for approximately 30 minutes before being loaded with boluses of the calcium-sensitive fluorophore Rhod-2 AM and the potentiometric fluorophore RH237 via a dye-injection port proximal to the aortic cannula. Simultaneous fluorescent signals of V_m_ and Ca^2+^ were recorded with 2 complementary metal oxide semiconductor (CMOS) cameras (RedShirt Imaging, Duluth, GA) with a sampling rate of 1 kHz and 34.4×34.4 mm field of view (128×128 pixels). To prevent motion artifact, cardiac contractions were arrested by treating the heart with the electromechanical uncoupler blebbistatin (Caymen Chemicals; 20 µM final concentration).

### Experimental protocol

Steady-state ventricular APs and CaTs were measured simultaneously during external pacing at 1, 3, and 5.5 Hz. To assess the sex differences in β-AR responsiveness, each heart was treated with increasing doses of the nonselective β-AR agonist Iso (Sigma): 1, 10, 31.6, 100, and 316.2 nM. Action potential duration (APD_90_) was defined as the time interval from the activation time to 90% of repolarization. Calcium transient duration (CaD_80_) was defined as the time interval from the time of the rapid rise of the CaT to 80% recovery of diastolic calcium levels. To quantify the rate of recovery of intracellular calcium to diastolic levels, the relaxation phase of the CaT (35–90% recovery of diastolic levels) was fitted with a single exponential decay function, and the decay time constant, τ (ms), was reported. Steady state parameter values were measured in the region of the LV base and LV apex. Spontaneous calcium release (SCR) events were defined as an unstimulated deflection in the diastolic Ca^2+^ signal that exceeded 10% of the amplitude of the full, paced beats in the preceding drive train. Instances of ectopic activity (ectopic beats, EBs) were identified from the ECG recording and defined as unstimulated beats that activated within 1.5 seconds from the end of the paced drive train. The thresholds for SCR and EB were defined in each heart as the dose of Iso (in nM) at which SCRs or EBs first occurred (i.e. the lowest dose of Iso that induced SCRs or EBs).

### Statistical analysis

Parameter values for a single experiment were derived from the mean value of a 5×5 pixel area of the area of interest (LV base or apex). Summary data are expressed as the mean of all experiments (mean ± SE). Differences in the means from male and female hearts were evaluated with two-factor ANOVA followed by two-tailed, unpaired Student t-tests. A two-tailed Fisher's exact test was used to test for a sex difference in the frequency of occurrence of SCR or ectopic activity. A two-tailed Mann-Whitney U test was used to compare the median dose thresholds for SCR or ectopic activity between female and male hearts. The significance level, α, was selected as 5%, therefore differences were considered to be statistically significant for p<0.05.

## Results

### Baseline sex differences in ventricular APs and CaTs

**Figure 2:**
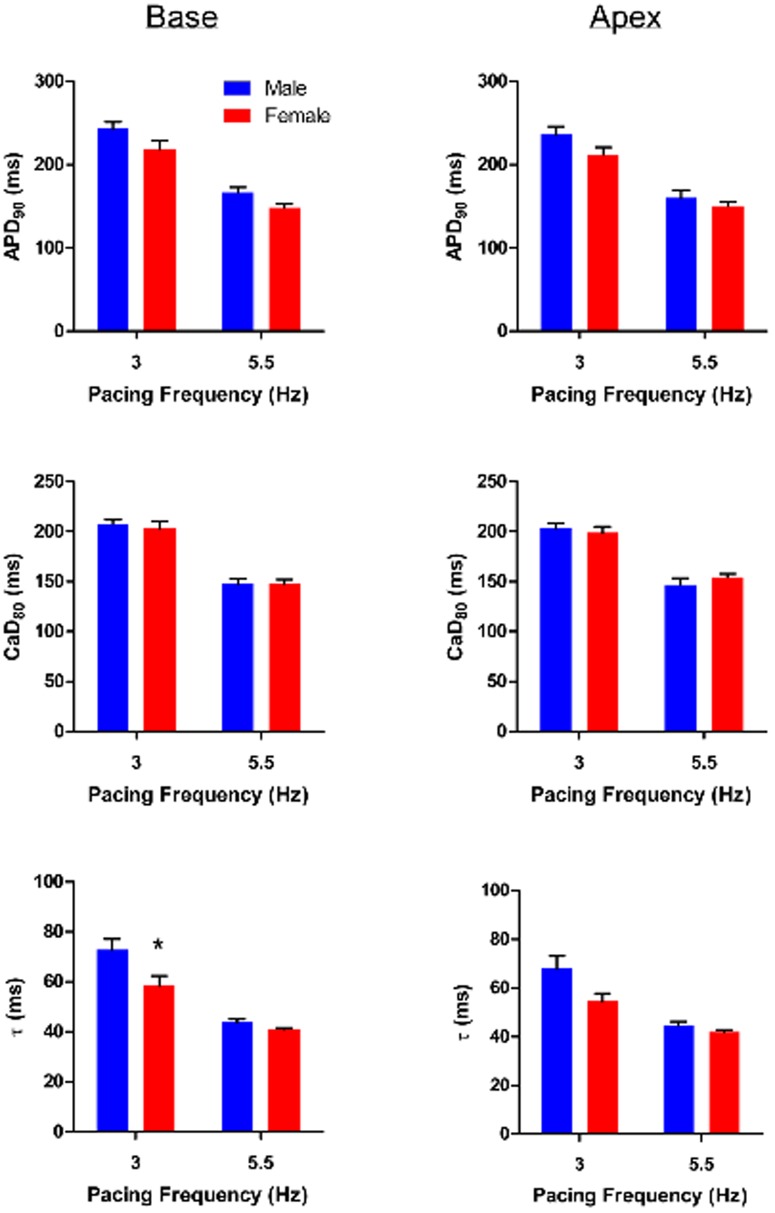
Baseline sex differences in steady-state ventricular APs and CaTs. Summary data for action potential duration (APD_90_, ms), calcium transient duration (CaD_80_, ms) and the time constant of calcium recovery (τ, ms) at 3 and 5.5 Hz pacing at either the LV base *(left)* or apex *(right)* in the absence of β-adrenergic stimulation (baseline). *p<0.05, female vs male.

Figure 2 (*top*) reveals that increasing the pacing rate from 3 to 5.5 Hz decreased APD_90_ at the LV base and apex in both male and female hearts (n = 5 male, 5 female). Interestingly, in the absence of β-AR activation there was a non-significant difference between APD_90_ from female hearts relative to male hearts. Specifically, female hearts had a mean APD_90_ of 218.6±10.0 ms (base) and 211.8±8.8 ms (apex) at 3 Hz, and 148.1±4.7 ms (base) and 150.1±5.1 ms (apex) at 5.5 Hz. Male hearts exhibited mean baseline APD_90_ of: 243.1±8.0 ms (base) and 236.2±9.4 ms (apex) at 3 Hz, and 166.5±6.2 ms (base, p = 0.06 vs female) and 160.2±9.1 ms (apex) at 5.5 Hz; which were not significantly different from the corresponding mean values in female hearts.

CaTs elicited under baseline conditions revealed similar calcium transient durations and sensitivity to pacing rate between the sexes. This is summarized in Figure 2 (*middle*) which demonstrates that there were no significant differences in CaD_80_ between male and female hearts at any pacing rate, but increasing pacing rate significantly shortened CaD_80_ in both regions of the heart (base and apex). Figure 2 (*bottom*) suggests that baseline sex differences in CaT decay kinetics are more marked at 3 Hz than 5.5 Hz. At 3 Hz, the mean CaT decay constant (τ) from the LV base was significantly faster (smaller τ) in female hearts than male hearts (58.5±3.9 ms versus 72.8±4.3 ms, p<0.05), while a similar trend existed for the LV apex (female  = 54.6±3.2 ms, male  = 68.0±5.1 ms, p = 0.06).

### Iso shortens APD_90_ in female hearts to a lesser extent than in male hearts

**Figure 3:**
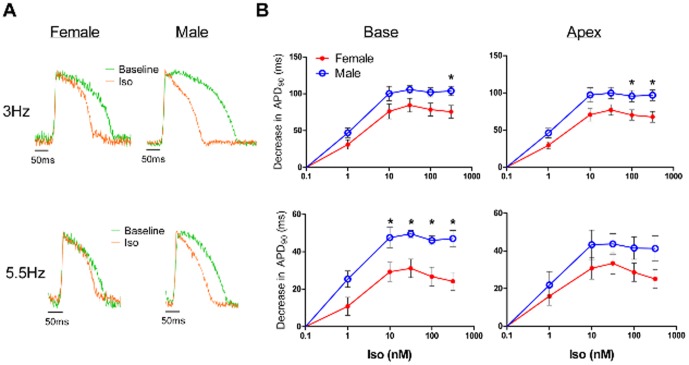
Less Iso-induced decrease of APD_90_ in female hearts. A) Representative APs at baseline (*green traces*) and during treatment with Iso (316.2 nM, *orange traces*) normalized and superimposed to demonstrate the sex difference in Iso effects during pacing at 3 Hz (*top*) and 5.5 Hz (*bottom*). B) Summary data for the decrease in APD_90_ (versus baseline values) as a function of the dose of Iso (1, 10, 31.6, 100, 316.2 nM) in the LV base (left) and apex of female (n = 5) and male (n = 5) hearts during pacing at 3 Hz (*top*) and 5.5 Hz (*bottom*). *p<0.05, female versus male.

Figure 3 shows the Iso-induced decrease in APD_90_ relative to baseline measurements as a function of the concentration of Iso for the LV base and LV apex at both 3 and 5.5 Hz pacing for male and female hearts. Shown in Figure 3A are superimposed APs from a single pixel at baseline (green) and with Iso (orange) in a female and male heart. From these representative APs, it is clear that there is a sex difference in the effect of Iso on APD_90_, with less APD_90_ shortening in the female AP than in the male AP. Iso shortened APD_90_ in a dose-dependent manner in all hearts, reaching a maximal effect at 31.6 nM Iso. Over all experiments, the decrease in APD_90_ was significantly less in female hearts than in male hearts, with sex differences seen at higher doses of Iso (Figure 3B, LV base: 316.2 nM at 3 Hz, 10–316.2 nM at 5.5 Hz; LV apex: 100–316 nM at 3 Hz; all p<0.05). These sex differences in the response to Iso were more pronounced at 5.5 Hz (versus 3 Hz) and in the LV base (versus LV apex). Overall, APD_90_ decreased 30–35% from baseline in female hearts and 40–45% in male hearts at 3 Hz; at 5.5 Hz, APD_90_ decreased 15–20% from baseline in female hearts and 25–30% in male hearts.

### No significant sex differences in CaD_80_ shortening in response to Iso

**Figure 4:**
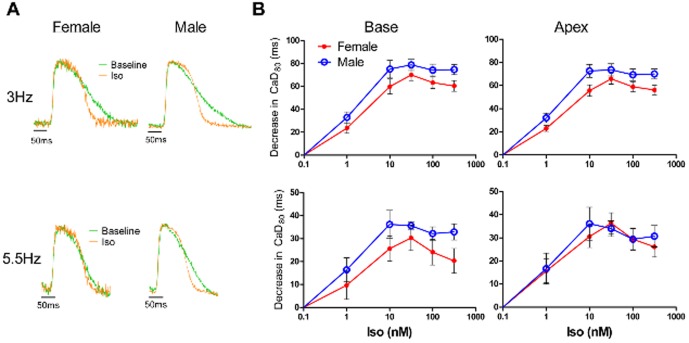
No significant sex differences in Iso-induced decrease of CaD_80_. A) Representative CaTs at baseline (*green traces*) and during treatment with Iso (316.2 nM, *orange traces*) normalized and superimposed to demonstrate the Iso-induced shortening of CaD_80_ at both 3 Hz *(top)* and 5.5 Hz *(bottom)* pacing. B) Summary data for the decrease in CaD_80_ (versus baseline values) as a function of the dose of Iso (1, 10, 31.6, 100, 316.2 nM) in the LV base *(left)* and apex *(right)* with pacing at 3 (*top*) and 5.5 Hz (*bottom*) for female (n = 5) and male (n = 5) hearts. Female versus male, p = NS for all comparisons.

Figure 4 shows the Iso-induced decrease in CaD_80_ relative to baseline measurements as a function of the concentration of Iso for the LV base and apex at both 3 and 5.5 Hz pacing for male and female hearts. Similar to APD_90_, treatment with Iso caused CaD_80_ to decrease in a dose-dependent manner, reaching a maximal effect at 31.6 nM Iso. The representative CaTs in Figure 4A show that Iso shortened the CaT from the female heart to a similar degree as in the male heart. The summary data in Figure 4B shows that while there was a trend for female hearts to have less of an Iso-induced shortening of CaD_80_ at 3 Hz (10 nM: apex, p = 0.05; 316.2 nM: base, p = 0.06, apex, p = 0.05), there were no statistically significant sex differences.

It was noted that the Iso-induced changes in CaD_80_ were more modest than the Iso-induced changes in APD_90_. To ensure that this “mismatch” was not merely a consequence of measuring APD at 90% repolarization versus CaD at 80% recovery, APD was also measured at 80% repolarization during treatment with 316.2 nM Iso. The mismatch was represented as the difference between the Iso-induced change (relative to baseline) in APD and CaD (ΔAPD80 – ΔCaD80), where positive values (in ms) represent greater Iso-induced changes in APD than in CaD. During 3 Hz pacing, the mismatch was 20.0±3.7 ms in the LV base and 17.8±4.5 ms in the LV apex. At the faster pacing rate (5.5 Hz), the mismatch was smaller (8.5±2.3 ms in the LV base and 5.0±2.8 ms in the LV apex). Thus the Iso-induced changes in APD were greater than those in CaD, independent of the level of AP repolarization.

### Iso decreases τ in female hearts to a lesser extent than in male hearts

**Figure 5:**
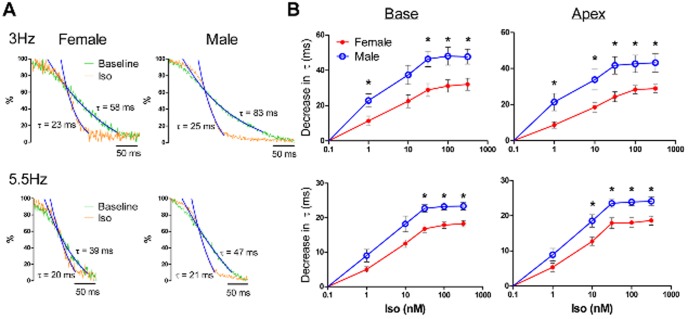
Less Iso-induced decrease of τ in female hearts. A) Relaxation phase of the CaT (peak amplitude to full recovery to diastolic levels) at baseline and during treatment with Iso superimposed to demonstrate the sex differences in the effect of Iso on the CaT decay kinetics at both 3 Hz *(top)* and 5.5 Hz *(bottom)* pacing. Single exponential decay functions (blue curves) were fit to the relaxation phase of the CaT, with the time constant of decay (τ) shown for each trace. B) Sex differences in the decrease in τ (versus baseline values) as a function of the dose of Iso (1, 10, 31.6, 100, 316.2 nM) in the LV base *(left)* and apex *(right)* of female (n = 5) and male (n = 5) hearts with pacing at 3 (*top*) and 5.5 Hz (*bottom*). *p<0.05, female versus male.

From the superimposed CaTs at baseline and with Iso shown in Figure 4A, it is apparent that the greatest difference between the baseline and Iso CaTs is during the CaT decline (particularly during late recovery); this strong lusitropic effect is reflected in the measurement of τ. In both male and female hearts, τ decreased as a function of Iso concentration up to 31.6 nM; at higher doses the lusitropic effect of Iso plateaued. In general, the Iso-induced decrease in τ was less in female hearts than in male hearts as evident in the superimposed relaxation phases from representative CaTs shown in Figure 5A. At 3 Hz (Figure 5B, *top*), these sex differences were significant for all doses of Iso tested except for 10 nM in the LV base (p = 0.05). At 5.5 Hz (Figure 5B, *bottom*), the Iso-induced decrease in τ was significantly less in females hearts versus male hearts for 31.6–316.2 nM in the LV base (1–10 nM, p = 0.07–0.08) and 10–316.2 nM in the LV apex.

### SCR and EBs during treatment with Iso

**Figure 6:**
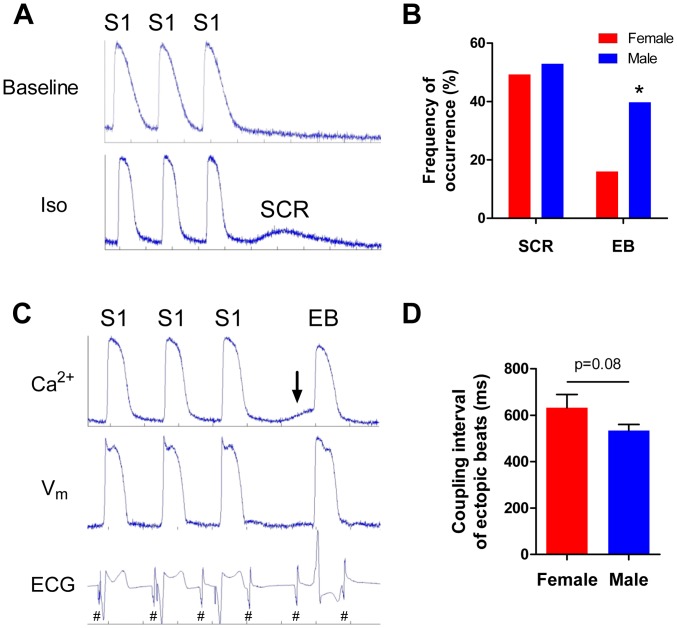
Iso-induced SCR and ectopic activity. A) Representative CaTs from a male heart during the last three paced beats (S1) of a 3 Hz drive train. *Top*, at baseline calcium levels return to diastole. During treatment with Iso (31.6 nM), an SCR occurs shortly after the return to diastolic calcium levels. B) Sex differences in the frequency of pacing protocols that elicited SCRs and EBs during treatment with Iso. C) Simultaneous recordings of Ca^2+^, V_m_, and the volume-conducted ECG from a male heart showing the last three paced beats (S1) followed by an SCR (arrow) and an EB elicited by treatment with 31.6 nM Iso. Note that the smaller, regularly occurring activations (#) in the ECG are derived from the intrinsic activity of the left atrium. This confirms the complete atrioventricular dissociation in CHB hearts. D) Sex difference in the coupling interval (ms) between the activation of the last paced beat and EBs elicited during treatment with Iso. *p<0.05, female vs male.

In addition to modulating APs and CaTs, it is known that β-AR activation can lead to spontaneous release of calcium from the sarcoplasmic reticulum (SR) during diastole and subsequent triggered activity. Therefore we wanted to determine whether or not the sex differences in the response to Iso observed in APD_90_ and τ were associated with sex differences in the occurrence of SCR or EBs. To elicit SCR activity and/or EBs, ventricular pacing was halted during the last 1.5–2 seconds of recording at each pacing frequency and dose tested (n = 5 male, 5 female). Figure 6A shows representative CaT traces from the last 3 paced beats (S1) before the halt in pacing at baseline and with Iso. At baseline, calcium declines to a resting level, but with Iso there is a subsequent increase in the diastolic calcium levels due to SCR. In some instances these SCR events were associated with the formation of an EB, as shown in Figure 6C.

No SCR or EBs were observed in any of the hearts (n = 10) in the absence of Iso (i.e. baseline). The threshold for SCR was 31.6 nM Iso in all hearts (male and female); interestingly, this is precisely the dose at which the maximal Iso response was reached in the dose response curves for APD_90_, CaD_80_, and τ (see Figures 3–5). There was no sex difference in the frequency of occurrence of SCRs during treatment with Iso (Figure 6B: female  = 37/75, male  = 36/68, p>0.20). However, EB formation occurred at higher doses of Iso in female hearts as opposed to male hearts, with a median dose threshold of 100 nM Iso for females and 31.6 nM Iso for males (p<0.05). Furthermore, Figure 6B shows that the frequency of EBs was significantly lower in female hearts (16% [ = 12/75]) than in male hearts (38% [ = 26/68], p<0.05). There was also a trend for the EBs in the female hearts to have a longer coupling interval (633±57 ms) than the EBs in the male hearts (534±27 ms, p = 0.08, Figure 6D).

## Discussion

The main findings of this study are that: 1) at baseline there were no significant sex differences in APD_90_ or CaD_80_, and only minimal differences in τ; 2) Iso reduced APD_90_, CaD_80_, τ in a dose-dependent fashion with “submaximal” effects observed at 1–10 nM Iso and “maximal” effects at ≥31.6 nM; 3) the Iso-induced changes in APD_90_ and τ (but not CaD_80_) were significantly less in female than male hearts, and these differences were enhanced during faster pacing rates (5.5 Hz) and higher doses of Iso (≥31.6 nM); 4) there were no sex differences in the frequency or dose threshold of SCR (31.6 nM); 5) however, the frequency of EBs was lower, and the dose threshold was higher in female than male hearts.

The present study provides novel information regarding sex differences in the dose response of APD_90_, CaD_80_, τ, and SCR/ectopic activity to the nonselective β-AR agonist Iso. Experiments were conducted at physiologic temperature (37°C) and pacing rates reflecting the physiologic range of HRs *in vivo* (3 and 5.5 Hz) in Langendorff-perfused hearts isolated from naïve, sexually mature rabbits of either sex. Experimental conditions which may impact the type or degree of sex differences observed include species, the type of preparation (e.g. isolated myocytes, intact tissue, whole hearts, or *in vivo*), regional variability of the tissue source (e.g. atria versus ventricle, base versus apex, transmural layer), temperature, stimulation frequency(s), age/development status of the animals, stage of the oestrus cycle of female animals (for species that have cyclical variations in sex hormone levels), and the type and dose(s) of sympathetic stimulation. All of these biological variables are likely to have a similar, or possibly even greater, impact on cardiac function as sex; failing to take these factors into account may confound any conclusions that are drawn.

Furthermore, the majority of studies investigating sex differences in β-adrenergic responsiveness have assessed the contractile response [8], [12]–[15]; a smaller number of studies have looked at calcium handling [16]–[18] or various ion currents [9], [10], [18]–[20] in isolated myocytes. However, there is a lack of studies addressing both electrical and mechanical cardiac function, thus the aim of these studies was to simultaneously image V_m_ and Ca^2+^ handling under physiologic conditions at steady-state and during protocols to elicit arrhythmogenic events associated with sympathetic stimulation.

### Baseline sex differences

Overall, few sex differences were found at baseline. In fact, in the absence of β-AR activation the only significant sex difference detected was a shorter decay constant (τ) in the CaTs from the LV base of female hearts during 3 Hz pacing. In the current studies, there were no sex differences in baseline APD_90_ from the base or apex of the LV epicardium at either 3 or 5.5 Hz pacing. While APD_90_ is commonly regarded to be longer in females than in males, such supporting data is typically from studies in isolated myocytes at room temperature with slow (non-physiologic) pacing rates; a closer review of the literature shows that studies are divided on whether APD or the QT interval in females is longer or similar to corresponding values in males (see Pham 2001 [21] for review). Studies conducted in intact tissue preparations at physiologic temperatures and pacing rates were more likely to show similar APDs in males and females [22]–[25]. To characterize ventricular CaTs, CaD_80_ was measured and the time constant (τ) of CaT decay was calculated. Similar to APD_90_, there were no sex differences in baseline CaD_80_. For τ, the sex-differences were rate-dependent, with shorter τ in females at 3 Hz, but no difference from males at 5.5 Hz. This difference was significant in the LV base, with a similar trend in the LV apex (p = 0.06). In rat CaTs, most studies have found that the CaT decay time is longer in females than in males [16], [26]. Not all studies saw longer CaT decay times in females though [27], and it has since been shown that this pattern reverses with age such that in young adults there are longer decay times in females but with aged adult rats the decay times are longer in males [28]. It is possible that age may influence the baseline sex differences in τ observed in the current studies.

### β-adrenergic responsiveness of APs

We found that the greatest sex differences in Iso-induced APD_90_ shortening (e.g. the separation of the male and female dose response curves) occurred at 5.5 Hz (Figure 3B). In the absence of β-AR stimulation, increasing the pacing rate from 3 to 5.5 Hz decreased APD_90_ to a similar degree in male and female hearts (Figure 2, *top*). The sex differences in Iso-induced APD_90_ shortening at 5.5 Hz may represent the reduced capacity of female hearts to further decrease APD_90_. Prior voltage clamp studies from our lab demonstrated that a smaller Iso-induced increase in I_Ks_ may underlie a reduced repolarization reserve in females (500 nM Iso increased I_Ks_ by 68% in isolated ventricular myocytes from naïve male rabbits, but only increased I_Ks_ in female myocytes by 19%) [20]. This study also suggested that much of the Iso-induced APD shortening in females was found to be independent of I_Ks_, possibly secondary to Iso-induced changes in calcium or sodium handling. The reduced β-AR responsiveness of I_Ks_ and APD in females could be due to altered expression of channel proteins such as KCNE1 [20], which is required for the β-AR effects on KCNQ1 current [29], or to reduced levels of cAMP as shown in female rat myocytes [9]. In addition, hearts from ovariectomized rats exhibit upregulation of β_1_-AR compared with hearts from control female rats [30], [31].

The ventricles of the heart are highly interconnected, forming a functional cardiac syncytium. Through the process of myocyte isolation, the cells are uncoupled from this syncytium which could potentially alter how these myocytes respond to β-adrenergic stimulation. Differences in pacing frequency and/or temperature may also explain varying degrees of sex differences in APD prolongation at baseline or with adrenergic stimulation in rabbit and other species [20], [32]–[34]. Finally, results from optical mapping of the epicardial surface the LV base and apex of Langendorff-perfused hearts may not be directly comparable to APs obtained from LV mid-myocardial myocytes [20] since APs from the epicardium and mid-myocardium have distinct profiles of repolarization [35], [36]. While the current study did not find striking differences in APD_90_ from the LV base and apex, APD_90_ was not measured in the mid-apicobasal region of the heart.

### β-adrenergic responsiveness of CaTs

In addition to providing novel data for sex differences in Iso-induced shortening of APD_90_ in intact ventricles under physiologic conditions, the current work also includes paired CaTs recorded simultaneously from the same locations as the APs. This capability allowed the detection of an apparent “mismatch” in the degree of AP and CaT changes induced by treatment with Iso. While Iso decreased APD_90_ to a lesser extent in female hearts (particularly at 5.5 Hz), the sex differences in Iso-induced decrease in CaD_80_ were not significant. In male hearts, the Iso-induced changes in APD_90_ were larger than the corresponding changes in CaD_80_ (see Figures 3 and 4) at both pacing frequencies. For example, at 5.5 Hz the maximum decrease in APD_90_ in the male hearts was 30% of the baseline value, but the maximum decrease in CaD_80_ from the paired CaTs was only 24% of baseline. A similar mismatch was observed in male and female hearts at 3 Hz. Interestingly though, due to the small decrease in APD_90_, this discrepancy between the Iso-induced change in APD_90_ and CaD_80_ was not present in female hearts paced at 5.5 Hz (maximum decrease in APD_90_ = 22%, CaD_80_ = 23%).

In addition to CaD_80_, the time constant (τ) of the relaxation phase was used to characterize the CaTs. In contrast to CaD_80_, the Iso-induced decrease in τ demonstrated significant sex differences with a much stronger lusitropic effect in male hearts than in female hearts. In a study using isolated rat ventricular myocytes, Chen et al. found no sex difference in the 50% decay time of CaTs either before or after treatment with Iso [17]. This discrepancy could reflect a species difference or a difference in early versus late calcium recovery as the 50% decay time (time from the peak of the CaT to 50% recovery) reflects early relaxation whereas τ, as defined in the current study (single exponential function fit to 35–90% of recovery), reflects the later portion of calcium recovery into the SR. Recovery of calcium into the SR is required to replenish SR calcium load, but the dramatic Iso-induced changes in τ have the potential to lead to SR calcium overload and subsequent spontaneous release of SR calcium (i.e. SCR).

### Iso-induced SCR and ectopic activity

Treatment of hearts with Iso led to the development of SCR (Figure 6). While there was no difference in the frequency of SCR occurrence in male and female hearts, these experiments established a dose threshold for SCR. All hearts (independent of sex) developed SCR activity at 31.6 nM Iso. This dose is of particular interest because it is precisely the dose of Iso at which the dose response curves for the decrease in APD_90_ and CaD_80_ are maximal, and where the curves for τ begin to plateau (see Figures 3–5). Despite the lack of sex difference in SCR occurrence, there was a lower median dose threshold and higher frequency of occurrence of EBs in male hearts than in female hearts. This suggests that SCR occurrence alone cannot account for ectopic activity and it is likely that other factors such as SCR amplitude and/or timing, or the “gain factor” of converting SCRs into delayed afterdepolarizations (DADs) contribute to the frequency of ectopic activity. As the sodium-calcium exchanger (NCX) is the primary source of the transient inward current responsible for DAD formation [37], [38], this finding could be potentially explained by sex differences in NCX expression or function. However, NCX current density was similar in male and female pig myocytes [19], and NCX protein expression was actually greater in females than in males for rat [14] and rabbit [39]. Further studies would be required to determine if other factors contribute to this phenomenon. Lastly, it was noted that there was a trend (p = 0.08) for the coupling interval between the activation of the last paced beat and the EB to be longer in female hearts than in male hearts, however additional EBs would need to be added to the analysis for sufficient statistical power. This finding is of interest because it is expected that EBs with shorter coupling intervals would be more likely to capture and initiate an arrhythmia.

## Conclusions

Iso decreased APD_90_, CaD_80_ and τ in a dose-dependent manner with maximal effects at approximately 31.6 nM, plateauing at higher doses. Overall, the Iso-induced changes in APD_90_ and τ (but not CaD_80_) were significantly less in female than male hearts. There were however, significant differences in the dose- and rate-dependence of the sex differences, thereby highlighting the importance of including these physiologically relevant variables in the experimental design. Submaximal effects were observed at lower doses of Iso (1–10 nM), which were associated with minimal sex differences. At higher doses (31.6–316.2 nM), the Iso effects were “maximal” (plateau in dose response curves) and the associated sex differences were enhanced relative to the lower doses. The regional heterogeneities between the base and apex of the LV were minimal, although greater sex differences were observed in the LV base at 3 Hz (versus the LV apex at 3 Hz). While no sex differences in the frequency of SCR activity were detected, these studies did establish a dose threshold for SCR activity which coincided with a plateau in the Iso dose response and the greatest sex differences in the response to Iso. In contrast, the frequency of EBs was lower, and the dose threshold was higher, in female hearts than male hearts; also, the coupling intervals of the EBs tended to be longer in female hearts. This could help explain the lower arrhythmia inducibility that has been observed in females. Altogether, the data demonstrate that female hearts had a decreased capacity to respond to β-AR activation, particularly under conditions of increased demand (i.e. faster pacing rates and “maximal” levels of physiologic β-AR activation), however this reduced β-adrenergic responsiveness of female hearts was also associated with less arrhythmic activity than in male hearts. These results provide a better understanding of the mechanisms of reduced β-adrenergic responsiveness in females and its cardioprotective effects, lay the foundation for future studies in our rabbit model of HF, and ultimately may lead to the development of sex-based treatment strategies.

## Supporting Information

Figure S1 **Iso-induced AP shortening for APD at 80% and 90% repolarization.** Decrease in APD (ΔAPD_X_) at 80% (APD80) or 90% (APD90) repolarization induced by treatment with 316.2 nM Iso (versus baseline) during pacing at 3 or 5.5 Hz in the LV base (Panel A) or apex (Panel B). *p<0.05 for male versus female.
(PDF)

Figure S2 **Iso-induced chronotropic effect.** Spontaneous HR in intact isolated hearts at baseline and change in HR during treatment with 100 nM Iso (ΔIso). No significant differences between female (n = 6) and male (n = 6) hearts.
(PDF)

Information S1 **Supporting information.**
(DOCX)
