# Supplementary figures and images for: Sex Differences in β-Adrenergic Responsiveness of Action Potentials and Intracellular Calcium Handling in Isolated Rabbit Hearts
Source: PLoS One. 2014 Oct 23;9(10):e111411. doi: 10.1371/journal.pone.0111411 (PMC4207827; doi:10.1371/journal.pone.0111411)

Figure S1

A

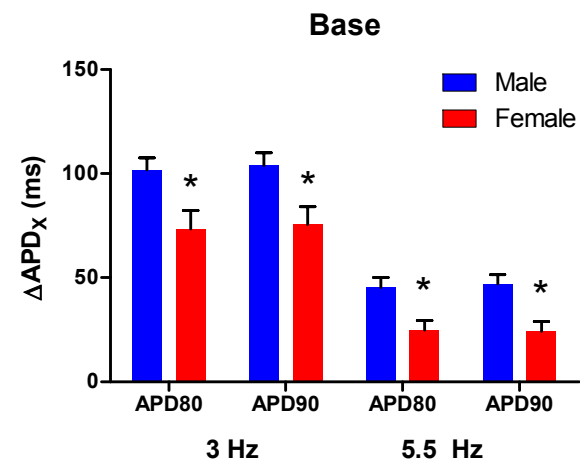

B

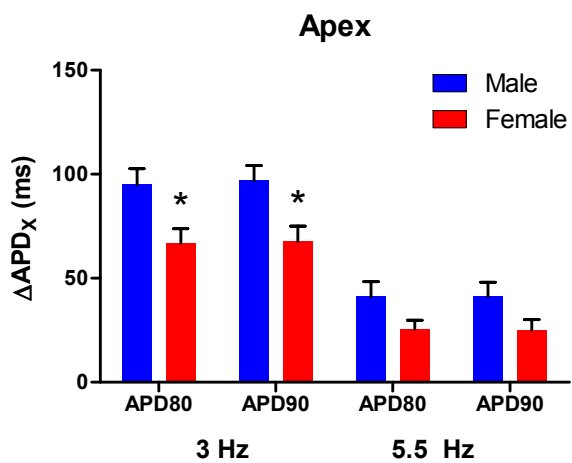

Supplement: Figure S1 — Iso-induced AP shortening for APD at 80% and 90% repolarization. Decrease in APD (ΔAPDX) at 80% (APD80) or 90% (APD90) repolarization induced by treatment with 316.2 nM Iso (versus baseline) during pacing at 3 or 5.5 Hz in the LV base (Panel A) or apex (Panel B). *p<0.05 for male versus female. (PDF) [file pone.0111411.s001.pdf]

Figure S2

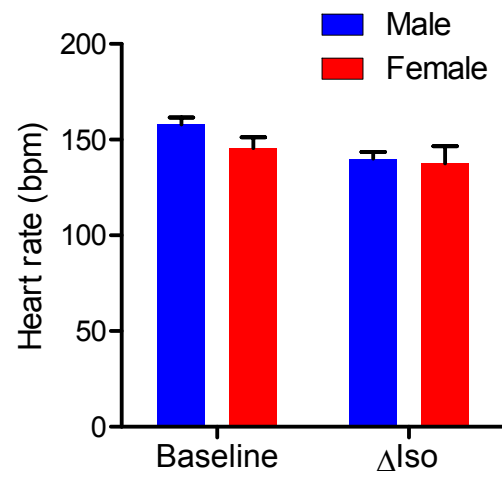

Supplement: Figure S2 — Iso-induced chronotropic effect. Spontaneous HR in intact isolated hearts at baseline and change in HR during treatment with 100 nM Iso (ΔIso). No significant differences between female (n = 6) and male (n = 6) hearts. (PDF) [file pone.0111411.s002.pdf]
