# Supplementary material for: Sex Differences in β-Adrenergic Responsiveness of Action Potentials and Intracellular Calcium Handling in Isolated Rabbit Hearts
Source: PLoS One. 2014 Oct 23;9(10):e111411. doi: 10.1371/journal.pone.0111411 (PMC4207827; doi:10.1371/journal.pone.0111411)
Supplement: Information S1 — Supporting information. (DOCX) [file pone.0111411.s003.docx]

**Supporting Information**

**Hoeker et al. Sex differences in β-adrenergic responsiveness of action potentials and intracellular calcium handling in isolated rabbit hearts.**

*Experimental preparation*

All experiments were conducted in compliance with the Guide for the Care and Use of Laboratory Animals published by the U.S. National Institutes of Health (NIH Publication No. 85-23, revised 1996). The protocols were approved by the Institutional Animal Care and Use Committee (IACUC) of the University of Alabama at Birmingham. Studies were conducted in naïve, sexually mature (age 6-9 months, 3.5-4.5 kgs) New Zealand White rabbits of both sexes (male n = 6, female = 6). Rabbits were purchased from either Millbrook or Harlan Labs; typically the rabbits were housed for an average of approximately 4 weeks prior to the date of study. Rabbits were preheparinized (2 ml heparin sulfate i.v., 1000 USP units/ml) and sedated with 44 mg/kg ketamine (intramuscular injection). The rabbits were then anesthetized with isoflurane (5%, mixed with 100% oxygen at 2 liters/min). Once in a surgical plane, the chest was opened and the heart was rapidly excised.

The aorta was cannulated and retrogradely perfused in Langendorff-fashion with a modified Tyrode’s solution (in mM: NaCl 128.2, KCl 4.7, NaHCO_3_ 20, NaH_2_PO_4_ 1.19, MgCl_2_ 1.05, glucose 11.1, CaCl_2_ 1.8, albumin 100 mg/L) that was heated and bubbled with 95% O_2_/5% CO_2_ to maintain physiologic temperature (36-37°C) and pH (7.40-7.45). The perfusate was filtered in-line with a nylon screen with a pore size of 11 μm. A peristaltic pump (MasterFlex L/S, Cole Parmer) was used to perfuse the heart; the perfusion pressure was monitored (BP-1 monitor with BLPR transducer, World Precision Instruments) and the flow rate was adjusted (typical range of 20-30 ml/min) to maintain a perfusion pressure of 50-70 mmHg. In preliminary studies it was found that treatment with 100 nM Iso dramatically increased the sinus rate from approximately 150 beats per minute (2.5 Hz) at baseline to nearly 300 beats per minute (5 Hz), thereby preventing the use of external pacing rates less than 5 Hz (see Figure S2). To address this issue, complete heart block (CHB) was induced by removing the right atrium (containing the sinus node) and ablating the atrioventricular node using electrocautery.[1] CHB resulted in complete atrio-ventricular dissociation and a junctional rhythm ≤ 60 beats per min, thereby allowing for testing of a wider range of pacing frequencies and to facilitate detection of ectopic beats. Two polytetrafluorethylene-coated silver wires (0.005 inch diameter) were inserted into the apex of the left ventricle (LV), spaced approximately 1-2 mm apart, to allow for endocardial pacing of the LV. The threshold for capture (in mA) was determined and LV pacing was conducted at twice diastolic threshold.

The heart was then mounted in a custom tissue bath (Figure 1) and immersed in the superfusate, which was heated to maintain a temperature of 36-37°C. The heart was positioned so that the anterior surface was visible through a viewing window (soda lime glass). Three evenly spaced electrodes mounted to the walls of the tissue bath were used to record a volume-conducted electrocardiogram (ECG) sampled at 1 kHz with a low frequency cutoff of 1 Hz, a high frequency cutoff of 3 kHz, and a 60 Hz notch filter. ECG, perfusion pressure, flow rate, and timing of the pacing stimuli were all recorded continuously throughout the entirety of the study, and synchronizations signals were embedded within the recording to mark time points of fluorescence recordings.

*Optical mapping system*

The isolated heart was allowed to stabilize for approximately 30 minutes before being loaded with boluses of the calcium-sensitive fluorophore Rhod-2 AM (Biotium; 0.5 mg dissolved in 0.5 ml DMSO [Sigma] and 0.5 ml PowerLoad [Invitrogen]) and the potentiometric fluorophore RH237 (Biotium; 30 μl aliquot of 2.52 mM stock solution dissolved in DMSO) via a dye-injection port proximal to the aortic cannula. To prevent motion artifact, cardiac contractions were arrested by switching the perfusate to a Tyrode’s solution containing the electromechanical uncoupler blebbistatin (Caymen Chemicals; 20 μM final concentration) and recirculated throughout the course of the experimental protocols.

Both fluorophores were excited with an array of six high-power green light emitting diodes (LEDs; Luxeon Rebel; 530 nm peak λ, typical luminous flux of 150 lm) fitted with bandpass excitation filters (Chroma Technology; 530/30 nm). The emitted fluorescence was collected by photographic grade lenses (Nikon, non-AF Nikkor lenses) with high numerical aperture, configured into a tandem lens assembly. The transmembrane potential (V_m_) and intracellular calcium (Ca^2+^) emission spectra were separated with a dichroic mirror (Chroma Technology; 630 nm LPXR, > 95% transmission) positioned at a 45° angle between the front and back lenses of the tandem lens assembly. Once split, the V_m_ and Ca^2+^ emission spectra passed through emission filters (Chroma Technology; V_m_: 695 nm longpass, Ca^2+^: 590/33 nm; transmission > 95% for their respective bandwidths of interest) before being focused on to two complementary metal-oxide-semiconductor (CMOS) detectors (RedShirt Imaging). Each CMOS camera has a 16.4 x 16.4 mm chip with 128 x 128 pixels, well depths up to 100 Me-, 21 bit dynamic range, and acquisition rates up to 2.5k frames per second. For this study the V_m_ and Ca^2+^ signals were each sampled at 1 kHz. The optical magnification was 0.48X, resulting in a pixel dimension of 0.27 mm and a field-of-view of 34.4 x 34.4 mm, which is approximately the size of the rabbit heart.

*Experimental protocol*

Steady-state ventricular APs and CaTs were measured simultaneously at the hearts’ intrinsic rate, as well as after 30 beat drive trains of external pacing at 3 and 5.5 Hz. To assess the sex differences in β-AR responsiveness, each heart was treated with increasing doses of the nonselective β-AR agonist isoproterenol (Iso; Sigma): 1, 10, 31.6, 100, and 316.2 nM. Iso was dissolved in double-distilled, deionized water and prepared as a stock solution (1 mM). The hearts were allowed to equilibrate at each dose for 4 minutes before running the pacing protocol. To assess the arrhythmogenic effect of β-AR activation, the hearts were paced at 1, 3, and 5.5 Hz and timed such that the last stimulus was delivered 1.5-2.0 seconds before the end of the recording. After the halt in pacing, the heart was monitored for any occurrences of spontaneous SR calcium release (SCR) or ectopy.

Post-processing and analysis of the fluorescence signals was carried out using a custom, Matlab-based analysis software package. Fluorescence signals were spatially binned (2x2) before further analysis. For optical APs, the activation time was defined as the maximum first derivative of the V_m_ signal (dF/dt_max_), corresponding to the steepest portion of the upstroke of the AP (phase 1). Action potential duration (APD_90_) was defined as the time interval from the activation time to 90% of repolarization. For optical CaTs, calcium transient duration (CaD_80_) was defined as the time interval from the time of calcium-induced calcium release from the SR (maximum first derivative of the Ca^2+^ signal, dF/dt_max_) to 80% recovery of diastolic calcium levels. To quantify the rate of recovery of intracellular calcium to diastolic levels, the relaxation phase of the CaT (35-90% recovery of diastolic levels) was fitted with a single exponential decay function, and the decay time constant, τ (ms), was reported. Steady state parameter values were measured in the region of the LV base and LV apex, identified from an image of the heart anatomy. SCR events were defined as an unstimulated deflection in the diastolic Ca^2+^ signal that exceeded 10% of the amplitude of the full, paced beats in the preceding drive train. Instances of ectopic beats (EBs) were identified from the ECG recording and defined as unstimulated beats that activated within 1.5 seconds from the end of the 30 beat paced drive train.

*Statistical analysis*

Parameter values for a single experiment were derived from the mean value of a 5 x 5 pixel area of the area of interest (LV base or apex). Summary data are expressed as the mean of all experiments (mean ± SE). Differences in the means from male and female hearts were evaluated with two-factor ANOVA followed by two-tailed, unpaired Student t-tests. A two-tailed Fisher’s exact test was used to test for a sex difference in the frequency of occurrence of SCR or ectopic activity. A two-tailed Mann-Whitney U test was used to compare the median dose thresholds for SCR or ectopic activity between female and male hearts. The significance level, α, was selected as 5%, therefore differences were considered to be statistically significant for p < 0.05.

*Assessment of the effect of motion artifact on APD measurements*

Sympathetic activation, as with Iso, increases contractility and may overcome the effects of excitation-contraction uncouplers, thereby producing motion artifacts that may interfere with the assessment of late AP repolarization times. To ensure that, if present, the effects of motion on the determination of APD_90_ were negligible, all calculations of APD_90_ were manually reviewed. Additionally, under the conditions in which motion is expected to be most severe (the highest Iso concentration, 316.2 nM), we measured APD at 80% repolarization (APD_80_) to verify that the choice of degree of repolarization did not affect the results. As shown in Figure S1, the Iso-induced changes in APD were consistent for both 80% and 90% repolarization and the significance of the sex differences in AP shortening in response to Iso were the same whether assessed by APD_90_ or APD_80_.

*Chronotropic response of intact hearts to isoproterenol*

At baseline, there was a trend for a slower sinus heart rate (HR) in intact female hearts (145.6 ± 5.7 bpm, n = 6) than in intact male hearts (157.9 ± 3.6 bpm, n = 6, p = 0.10), but this difference did not reach statistical significance (Figure S2). In intact hearts, treatment with 100 nM Iso nearly doubled the sinus HR of spontaneously beating isolated hearts (Figure S2). There was no sex difference in the HR increase in response to Iso (female ΔHR = 96.1 ± 8.9%, male ΔHR = 93.3 ± 2.8%; p = NS).

**References**

1. Nemec J, Kim JJ, Gabris B, Salama G (2010) Calcium oscillations and T-wave lability precede ventricular arrhythmias in acquired long QT type 2. Heart Rhythm 7: 1686-1694.
